# Health-Related Quality of Life and Associated Factors in Patients with Atrial Fibrillation: An Integrative Literature Review

**DOI:** 10.3390/ijerph16173042

**Published:** 2019-08-22

**Authors:** Youn-Jung Son, Kyoung-Hwa Baek, Suk Jeong Lee, Eun Ji Seo

**Affiliations:** 1 Red-Cross College of Nursing, Chung-Ang University, Seoul 06974, Korea; 2 Division of Nursing, Gyeongbuk College of Health, Kimcheon-Si 39525, Korea; 3 Ajou University College of Nursing and Research Institute of Nursing Science, Suwon 16499, Korea

**Keywords:** atrial fibrillation, health-related quality of life, integrative review

## Abstract

Atrial fibrillation (AF) is a common cardiac arrhythmia associated with poor health-related quality of life (HRQoL). However, the factors influencing HRQoL in patients with AF are not well understood. The purpose of integrative review was to investigate the factors affecting HRQoL in patients with AF based on the six domains of Ferrans and colleagues’ HRQoL model. A total of 23 relevant articles published between January 2000 and March 2018 were identified using four databases and analyzed in this study. Our review showed that the HRQoL in patients with AF was consistently lower than both healthy individuals and patients with other cardiovascular diseases. The most common factor associated with HRQoL in patients with AF was anxiety-specific to AF in the symptoms domain, followed by frequency and severity of symptoms and the New York Heart Association functional class. This study highlights that monitoring and assessing patients’ symptoms is vital for improving HRQoL in patients with AF. Disease-specific and cross-culturally validated tools can allow healthcare professionals to provide tailored interventions for patients with AF.

## 1. Introduction

Atrial fibrillation (AF) is a common supraventricular arrhythmia which affects approximately 1% to 4% of the global population. AF is more common in older individuals; the prevalence of AF increases sharply in those over 80 years old. Approximately 10% of individuals in this age group are affected with AF [1,2]. AF has a broad range of symptoms, with 25–30% of patients experiencing no noticeable symptoms [3]. Patients with AF are five times more likely to have a stroke and three times more likely to experience heart failure compared to healthy individuals [4]. AF may also increase an individual’s socioeconomic burden through increased healthcare costs [5].

In managing AF, anticoagulant treatment is important for preventing stroke. However, the fear of complications associated with treatment, such as bleeding, can isolate patients from social activities, leading to a decreased health-related quality of life (HRQoL) [1]. HRQoL is a personal perception of the state of life in relation to personal goals or expectations. Thus, emphasizing HRQoL is important when assessing the overall health of patients with AF [6]. Previous studies reported that HRQoL is significantly impaired in patients with AF compared to the general population and patients with structural heart diseases [7,8]. However, there is controversy over the effects of sociodemographic (sex, level of activities), clinical (ejection fraction, comorbidity, stroke risk score, treatment methods), and psychological factors (stress, anxiety) on HRQoL in patients with AF [9,10,11]. Previous systematic reviews only covered symptom severity, HRQoL instruments used, and changes in HRQoL after rate/rhythm-control interventions [7,8,9,12]. Therefore, more investigation into the factors associated with HRQoL in patients with AF is necessary.

The integrative review method includes more diverse methodologies than a systematic literature review and therefore can present varied perspectives on a phenomenon of the subject in the original research and strengthen the overall understanding of the subject [13]. Existing literature on HRQoL in patients with AF includes quantitative and qualitative research. Additionally, the scope of outcomes in the interventional study included in this analysis is narrow due to the inclusion of patients with various disease characteristics. Therefore, an integrative review is appropriate to combine the heterogeneous body of existing research and improve the overall understanding of HRQoL in patients with AF [14].

Improving HRQoL is a major goal in the management of AF, however, it is still not well understood. Thus, this study examines the HRQoL-related factors using the well-known HRQoL model developed by Ferrans and colleagues as the conceptual framework [15]. Ferrans and colleagues’ model consists of six domains: Biological function, symptoms, functional status, general health perceptions, characteristics of the individual, and characteristics of the environment [15]. It provides clear conceptual and operational definitions and clarifies relationships among concepts to guide both research and practice [16]. Using this model, we comprehensively explored the factors related to HRQoL in patients with AF.

## 2. Materials and Methods

### 2.1. Study Design

**Figure 1:**
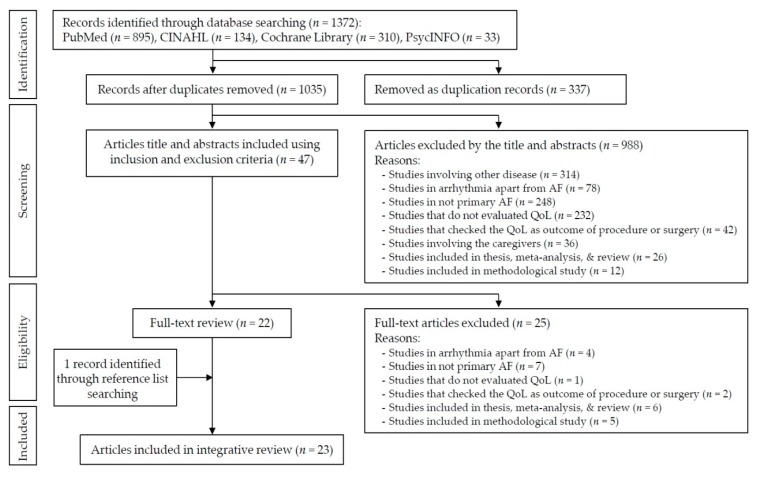
Preferred Reporting Items for Systematic Reviews and Meta-Analyses (PRISMA) flow diagram of the literature search and study selection process.

This review was conducted using Whittemore and Knafl’s methodology for integrative review [13]. This methodology is useful for a holistic understanding of multiple perspectives. The stages for integrative review included: Problem identification, literature search, data evaluation, data analysis, data interpretation, and presentation of results. The flow diagram of the identified literature is presented using the Preferred Reporting Items for Systematic Reviews and Meta-Analyses (PRISMA) [17] (Figure 1).

### 2.2. Problem Identification and Literature Search

A literature search was conducted to identify relevant articles published in English between 1 January 2000 and 31 March 2018 using the PubMed, Cumulative Index to Nursing and Allied Health Literature (CINAHL), Cochrane Library, and PsycINFO databases. The Medical Subject Headings and search terms used alone or in combination were as follows: (1) “Atrial Fibrillation” or “Paroxysmal Atrial Fibrillation” or “Persistent Atrial Fibrillation”, and (2) “Quality of Life” or “Health related Quality of Life”, or “HRQoL”. Additionally, ancestry searches or backward searches were conducted on all eligible primary studies. The search was limited to publications in peer-reviewed journals. We conducted a preliminary assessment of the titles and abstracts of identified studies and then assessed the full text of relevant articles in detail. The inclusion criteria were the following: (1) published in peer-reviewed journals, (2) focus on patients with AF, (3) concern HRQoL as dependent or independent variables, (4) be written by English, and (5) have been published between January 2000 and March 2018. The specific year was chosen because HRQoL began to be properly assessed in general patients with AF in 2000 [18,19]. We excluded non-original research, study protocol, development of instrument, non-human results, and HRQoL as an outcome for pharmacological and electrical interventions. Two authors (Y-J.S. and K-H.B.) independently screened the studies according to the criteria and discussed the screening results. Finally, the 23 papers met the criteria to be included in this review. Figure 1 shows the complete process.

### 2.3. Data Evaluation

The Mixed Methods Assessment Tool (MMAT) was used to evaluate the quality of studies with different study designs [20]. This critical appraisal tool has the advantage of being able to evaluate quantitative, qualitative, and mixed research methods studies. Recently, this tool has been used for quality evaluation in integrated literature review [21]. Quality is scored on a scale from 0% (never consistent with the standard) to 100% (fully consistent with the standard) [20].

The 23 included studies [22,23,24,25,26,27,28,29,30,31,32,33,34,35,36,37,38,39,40,41,42,43,44] were evaluated using the MMAT. The two screening questions applied to all reviewed studies regardless of study design. Afterwards, we selected and answered appropriate criteria according to each study design. Two authors (Y-J.S. and E.J.S.) independently evaluated the studies according to the criteria and discussed the results.

All 23 studies satisfied the two screening questions. The two qualitative studies satisfied all quality appraisal criteria, which had superior methodology. In the three randomized control studies, the quality appraisal criteria were 100% satisfied for one study. Two studies did not satisfy randomization and allocation concealment. Among the 18 descriptive studies, nine studies were 100% appropriate, five studies were 75% appropriate, and four studies were 50% appropriate. Most of the descriptive researches satisfied the following questions: “Is the sampling strategy relevant to address the research question?” and “Are measurements appropriate?” However, nine of the descriptive studies did not satisfy the question “Is there an acceptable response rate (60% or above)?”

### 2.4. Data Analysis

Two researchers read and analyzed each study independently. They met eight times for review, data reduction, data display, data comparison, and conclusion extraction. The characteristics and results of the studies are summarized in Table 1. The type of AF was analyzed according to the AF criteria in the American College of Cardiology and the American Heart Association guideline [1].

The factors affecting HRQoL were analyzed according to the HRQoL model from Ferrans and colleagues [15]. Ferrans and colleagues’ model has six domains: Biological function, symptoms, functional status, general health perceptions, characteristics of the individual, and characteristics of the environment. The biological function domain assesses the function of cells, organs, and systems through laboratory tests, physical assessment, and medical diagnoses. The symptoms domain includes physical, emotional, and cognitive symptoms as perceived by the patient. The functional status domain examines physical, psychological, social, and role functioning through evaluation of the remaining level of function. The general health perceptions domain is a subjective rating and opinion on their health. Characteristics of the individual refer to demographics that influence health outcomes, such as age, sex, responsiveness, and body mass index. Characteristics of the environment are categorized as social factors (such as family and healthcare providers) or physical factors (such as home and workplace).

## 3. Results

### 3.1. Characteristics of Included Studies

Table 1 shows the general characteristics of the 23 included studies. In total, 13 of the 23 studies were conducted in Europe or the United States. Regarding research design, there were three randomized controlled trials, nine cross-sectional studies, five prospective studies, four case-control studies, and two phenomenological studies of qualitative research. The study participants were outpatients in 16 studies, inpatients in five studies, and unidentified in two studies. The sample sizes ranged from 15 to 10,087 and the average patient age was between 53.2 and 75.0 years. The type of AF was described in 19 studies.

### 3.2. HRQoL Scores in Patients with AF

The mean HRQoL as evaluated by the Short Form 36 health survey (SF-36) was 36.6–46.0 (physical composite summary, range 0–100) and 40.8–55.0 (mental composite summary, range 0–100). The mean HRQoL as evaluated by the symptom checklist was 14.0–22.0 (symptom frequency, range 0–64) and 12.0–19.0 (symptom severity, range 0–48). The HRQoL in patients with AF was consistently lower than either healthy individuals or patients with other cardiovascular diseases (Table 1).

The mean HRQoL as evaluated by AF-specific instruments was 59.5–89.8 (atrial fibrillation effect on quality of life, range 0–100) and 7.11–13.5 (University of Toronto atrial fibrillation severity scale, range 0–35). One study using the atrial fibrillation quality of life questionnaire (AFQLQ) presented an AFQLQ1 score of 15.7 (frequency of symptom, range 0–24), AFQLQ2 score of 12.3 (severity of symptom, range 0–18) and AFQLQ3 score of 45.0 (limitation and anxiety, range 0–56).

### 3.3. Characteristics of the Instruments for HRQoL in Patients with AF

In the 23 studies, 13 different HRQoL instruments were used (Table 2). General, cardiac-specific, and AF-specific instruments were used either together or individually. SF-36 was used most frequently (15 studies). AF- and cardiac-specific instruments were each used for six studies. The reliability and validity were reported for all 13 HRQoL instruments except the EuroQol-visual analogue scale.

### 3.4. The Factors Influencing HRQoL in Patients with AF Based on HRQoL Model

A total of 32 variables were presented in the 23 articles as influencing factors for HRQoL in patients with AF. We organized the variables into six domains based on Ferrans and colleagues’ model [15] (Table 3). The most common factor influencing HRQoL in patients with AF was anxiety related to AF (four articles). This was followed by the frequency and severity of symptoms and New York Heart Association (NYHA) functional class.

The biological function domain included nine variables, such as illness duration, left ventricular function, CHADS_2_ score as a stroke risk stratification system [1], and brain natriuretic peptide level as marker for heart failure [45]. The symptom domain included anxiety, symptom frequency and severity, depression, stress, and uncertainty. Frequent symptoms, severe symptoms, and negative psychological conditions were related to a low HRQoL. In the functional status domain, a high NYHA functional class or low exercise performance level were related to a low HRQoL. The general health perceptions domain included illness perception and fear of AF. HRQoL was high when the general perception about the disease was positive, but fear of AF attack significantly reduced HRQoL. In two qualitative studies [34,42], the patients’ perception on AF was reported without the associated statistical significance. All characteristics of the individual reported in reviewed studies were significantly related to HRQoL. The characteristics of the environment, including financial status and whether or not patients were involved in significant relationships, were related to HRQoL.

## 4. Discussion

This integrative review investigated the factors related to HRQoL in patients with AF. Based on Ferrans and colleagues’ model [15], factors influencing HRQoL were categorized into six domains: Biological function, symptoms, functional status, general health perceptions, characteristics of the individual, and characteristics of the environment. Importantly, this review showed that symptoms domain was the most important on HRQoL in patients with AF. Specifically, anxiety and symptom frequency/severity in the symptom domain were the most frequently reported factors influencing HRQoL in patients with AF. In previous studies, symptoms such as anxiety, depression, perceived stress, and uncertainty were significantly associated with HRQoL among patients with AF [1,3,7]. Sudden and unpleasant symptoms were also a key theme in the qualitative studies included in this review [34,42]. Major emotional burdens on patients with AF include symptoms, concerns regarding complications, and uncertainty about the future [46]. Therefore, healthcare professionals should provide the interventions that explain the current status and complications risk of individual patients and guide the management strategies for each patient in detail. These proactive approaches could reduce patients’ physical and psychological symptoms and help manage HRQoL for patients with AF.

The biological function domain is the pathophysiological change in individuals with AF. This change is an objective mechanism that can explain the health status based on the patient’s specific condition and identify the characteristics of their disease [47]. Improvement of the biological function is a starting point for holistic care [15]. Healthcare professionals should therefore understand the importance of periodically assessing physical health through cardiac-specific laboratory tests and physical examinations. According to this review, however, there was still controversy over the influence of the biological factors, such as duration of illness and left ventricular function, on HRQoL. Therefore, further studies should be carried out to better understand the variables in the biological function domain.

The general health perception, such as the patients’ acceptance of their disease, was also related to HRQoL in this review. Interventions that can change patients’ perceptions may have a multifaceted, direct effect on HRQoL as perception can affect all aspects of individuals [6]. However, our findings could not specify the significance of most predictors in the general health perception domain because the few studies available were qualitative. Therefore, more research is needed to better understand patients’ perceptions of AF itself, its effect on daily life, and the associated burdens, as well as the practical effects of their perceptions.

As a result, the HRQoL of patients with AF could be improved with better control of symptoms and a better understanding of individual pathophysiological changes. Additionally, a positive perception of AF could be formed through patient-centered explanations of treatment and complications, which could further improve HRQoL.

This review analyzed a broad body of research on the HRQoL in patients with AF. One possible explanation for the observed variability in results could be the variability of characteristics in patients with AF. Although AF is one disease, it has long been recognized that different subtypes of AF are associated with different risks of complications [48]. In planning patient-centered interventions, these findings emphasize the importance of identifying the multi-dimensional factors influencing HRQoL. This will help target key domains of HRQoL, which may differ based on the specific patients’ characteristics [10,49]. Despite these variations, this review showed the overall HRQoL of patients with AF was lower than that of healthy individuals and other patients with cardiac diseases, which is in line with the results of previous studies [7,8].

In this review, most studies used generic HRQoL instruments such as SF-36 instead of disease-specific tools. In general, disease-specific instruments are substantially more responsive than generic instruments and may therefore be more suitable to assess the disease’s impact on HRQoL [50]. Therefore, it is necessary to develop a reliable and valid AF-specific instrument that can reflect the reality of patients with AF and the influencing factors based on a common HRQoL model.

This review has several limitations. First, AF populations were heterogeneous because samples in the studies included in this review were recruited from either primary-care or tertiary-care institutions. This makes generalizing the results difficult because most patients referred to tertiary clinics have a greater disease burden than the average AF patient. Second, many of the studies included in this review were cross-sectional observation studies. AF is a chronic disease that requires continued treatment and self-care. Therefore, HRQoL should be monitored continuously through prospective studies, which can examine changes of HRQoL over time. In addition, further research is needed to confirm causal relationships between identified influencing factors and HRQoL in patients with AF. Third, there were studies in this review that reported characteristics of patients with AF that were not controlled during the statistical analysis. Patient characteristics have an important effect on HRQoL, and therefore the characteristics should be fully accounted for in future studies. Lastly, we did not search all grey literature databases (e.g., searching dissertation databases) or internet-based search engines such as Google or Google Scholar.

## 5. Conclusions

This review showed that AF-related anxiety, symptom frequency, and symptom severity were associated with a poor HRQoL. Thus, controlling symptoms and preventing complications such as stroke and heart failure should be considered to improve the HRQoL of patients with AF. Anxiety, the most common factor affecting HRQoL, could be reduced through patient-oriented explanation of their current status and health management strategies. Therefore, healthcare professionals must monitor patients’ perceptions about AF, anxiety level, stroke/bleeding risk, and biological function to ensure early intervention. A reliable AF-specific HRQoL instrument based on multi-dimensional patient characteristics should be developed and validated to design patient-centered interventions.

In this review, Ferrans and colleagues’ model provided an organized approach to identify the various influencing factors for HRQoL and to understand the relationships between factors. It is the strength of this review. Use of HRQoL model will provide more evidence about which relationships among HRQoL concepts are common to different populations. Therefore, our methodology can be a guide to further study for factors of HRQoL in other chronic diseases.

## Figures and Tables

**Table 1:** Descriptive summary of main characteristics and results of studies (*n* = 23).

Author (year), Country	Study Design	Admission Type	Sample Characteristics		Main Findings	Quality Appraisal by MMA (%)
			AF Type	*n*, male%, Mean Age (yrs)		
Dorian et al. (2000), Canada [22]	Case-control	Outpatients	Paroxysmal Persistent	AF; 152, 73%, 58.0 Healthy; 47, 45%, 54.0 PTCA; 69, 79%, 62.0	The SF-36 subscale scores were lower in AF patients compared to the PTCA, healthy group (*p* < 0.05). Case group reported worse QoL than PTCA and healthy groups.	100
Suzuki et al. (2004), Japan [23]	Cross-sectional	Outpatients	Paroxysmal	240, 69.6%, 57.9	A significant difference was found between agoraphobic patients and nonagoraphobic patients in SDQL. Psychological stress is the main perceived inducer in daily life, and attack induced by psychological stress affects their anxiety symptoms and QoL.	50
Van den Berg et al. (2005), Netherlands [24]	Cross-sectional	Outpatients	Paroxysmal	73, 68.5%, 55.5	QoL in the physical domain and pain was not related to the degree of neuroticism (*p* = 0.81).	50
Maryniak et al. (2006), Poland [25]	Cross-sectional	Inpatients	Paroxysmal	76, 73%, 53.2	No significant relationship was shown among disease duration, comorbidities, and QoL.	50
Ong et al. (2006), Canada [26]	Cross-sectional	Outpatients	Paroxysmal Persistent Permanent	93, 66%, 61.8	PCS was 45.31 and MCS was 52.52 (SF-36: 0–100). AFSS was 7.11 (AFSS: 0–35)	75
Singh et al. (2006), USA and Canada [27]	Randomized controlled trial	Outpatients	Persistent	Con; 305, 99%, 67.3 Exp; 319, 99.7%, 66.4	Favorable changes were seen in SF-36 subscales in SR patients at 1 year in general health (*p* = 0.007) and social functioning (*p* = 0.002).	50
Hegbom et al. (2007), Norway [28]	Randomized controlled trial	Outpatients	Chronic	Con. 15, 86.7%, 64.0 Exp. 13, 100%, 62.0	SF-36 subscales (physical functioning, bodily pain, vitality, and role-emotional) improved significantly following Exercise Training Program.	50
Baek et al. (2008), Korea [29]	Cross-sectional	Outpatients	Persistent Permanent	102, 55.9%	QoL measured by SF-36 (range: 0–100) had a low to moderate correlation with symptom frequency and severity. There were no significant differences in QoL according to gender, taking aspirin, or taking warfarin.	100
Kang (2009), Korea [30]	Cross-sectional	Outpatients	Not mentioned	129, 50.3%, 63.2	Americans’ QoL measured by SF-36 (range: 0–100); 33.53/51.43 vs. 41.46/46.12 (physical function/mental health, female vs. male) Koreans’ QoL measured by SF-36 (range: 0–100); 36.62/41.24 vs. 45.44/49.32 (physical function/mental health, female vs. male) The significant interaction effect of gender and culture on mental health was shown.	75
Lane et al. (2009), UK [31]	Prospective cohort	Outpatients	Persistent Permanent	70, 64.3%, 71.4	There were no significant differences in the levels of depression and perceived stress and HRQoL (except for an increase in energy and decline in general health perception) over the 12 months following diagnosis. Illness identity and beliefs about medication are significant predictors of the improvement in physical HRQoL over time.	100
Dabrowski et al. (2010), Poland [31]	Case-control	Not mentioned	Paroxysmal Persistent Permanent	AF; 95, 63.3%, 67.8 Healthy; 70, 42.9%, 55.5	The scores of NHP (range: 0–100) were lower in paroxysmal, persistent, and permanent AF patients compared to the healthy.	100
Jaber et al. (2010), Brazil [33]	Case-control	Outpatients	Chronic	89, 100%, 54.2	There was a significant difference in QoL in physical and mental summary scores in patients with maximal HR ≤ 110 bpm on 6MWT in comparison with HR > 110 bpm and in the physical summary score in patients with average HR ≤ 80 bpm on Holter monitor in comparison with HR > 80 bpm.	75
McCabe et al. (2011), USA [34]	Phenomenology	Outpatients	Paroxysmal Persistent	15, 53.3%, 59.8	Themes included (1) finding the meaning of symptoms, (2) feeling uninformed and unsupported, (3) turning points, (4) steering clear of AF, (5) managing unpredictable and function-limiting symptoms, (6) emotional distress, and (7) accommodation to AF tempered with hope for a cure.	100
Dorian et al. (2013), Canada [35]	Prospective cohort	Inpatients	Paroxysmal Persistent Permanent	210, 56.7%, 62.1	AF patients’ QoL measured by AFEQT (range: 0–100). 59.5 (baseline); 72.4 (3 months) (*p* < 0.01) The improvement that 19 points in the AFEQT score changed can be termed a meaningful, important improvement.	100
Goren et al. (2013), USA [36]	Case-control	Outpatients	Not mentioned	1,296, 65.1%, 64.9	AF patients’ vs. non-AF controls’ QoL measured by SF-36 (range: 0-100); 38.6 vs. 44.8 (*p* < 0.001, PCS), 49.7 vs. 51.6 (*p* < 0.001, MCS) AF patients had lower MCS, PCS and utility scores, greater activity impairment, more traditional provider visits, and increased emergency room visits and hospitalizations.	75
Lakkireddy et al. (2013), USA [37]	Prospective cohort	Outpatients	Paroxysmal	49, 46.9%, 60.6	Yoga training improved the QoL parameters of physical functioning, general health, vitality, social functioning, and mental health domains on SF-36.	100
Lee et al. (2013), Korea [38]	Cross-sectional	Inpatients	Paroxysmal Persistent	150, 51.3%, 62.4	PCS was 38.92 and MCS was 41.49 (SF-36: 0–100). Physical and mental HRQoL had significant correlations with uncertainty, anxiety, and depression.	100
Schron et al. (2014), USA [39]	Cross-sectional	Not mentioned	Not mentioned	693, 62.2%, 69.8	History of stroke, heart failure, rhythm control, lower QoL (PCS and MCS in SF-36) predicted hospitalization. Diabetes, female gender, older age, CAD, hypertension, and lower PCS in SF-36 predicted mortality.	75
Tsounis et al. (2014), Greece [40]	Cross-sectional	Inpatients	Paroxysmal Persistent Permanent	108, 64%, 65.4	PCS was 40.28 and MCS was 40.89 (SF-36, range: 0–100). EQ-VAS (range: 3–100) was 59.63.	50
Yamamoto et al. (2014), Japan [41]	Prospective cohort	Outpatients	Paroxysmal	233, 71%, 64.9	Asymptomatic AF episode frequency correlates with a reduced QoL in patients with paroxysmal AF.	100
Altiok et al. (2015), Turkey [42]	Phenomenology	Outpatients	Not mentioned	32, 50%, 66.9	Four main themes and 15 subthemes were identified: (1) patient’s mental status regarding the disease, (2) patient’s social status regarding the disease, (3) patient’s physical condition regarding the disease, and (4) disease management and coping with the disease.	100
Freeman et al. (2015), USA [43]	Prospective cohort	Outpatients	Paroxysmal Persistent Permanent	10,087, 57.6%, 75	The AFEQT score decreased with increasing EHRA symptom severity class. Lower QoL was associated with a higher risk of hospitalization, but not other major adverse events, including death.	100
Bowyer et al. (2017), Australia [44]	Randomized controlled trial	Inpatients	Paroxysmal Non-paroxysmal	Con; 19, 57.9%, 62.1 Exp; 22, 76.2%, 61	The nurse intervention group showed significant differences compared to the control with respect to higher QoL on the SF-36 score of physical functioning and vitality at six months.	100

For all reported scores, a higher score indicates a worse QoL (quality of life) in the NHP (Nottingham Health Profile) but a better QoL in others. Higher score indicates more frequent and serious symptoms. AF: Atrial fibrillation; AFEQT: Atrial fibrillation effect on quality of life; AFQLQ: Atrial fibrillation quality of life questionnaire; AFSS: University of Toronto atrial fibrillation severity scale; Con: Control group; EQ-5D: Euroqol-5d; EQ-VAS: Euroqol-visual analog scale; Exp: Experimental group; HR: Heart rate; HRQoL: Health-related quality of life; IIRS: Illness intrusiveness rating scale; MMA: Mixed methods appraisal; MWT: Minute walk test; PCS: Physical component summary; PTCA: Percutaneous transluminal coronary angiography; QLI-CV: Quality of life index-cardiac version; SAS: Specific activity scale; SCL: Symptom checklist; SDQL, Scale of disease and quality of life; SF-36: Short form 36 health survey; SR: Sinus rhythm, yrs: Years.

**Table 2:** Instruments used to measure HRQoL in patients with AF in reviewed studies.

Instrument Type	Instrument	Article Number	No. of Items	Reliability *	Validity
AF-specific	AFEQT	[35,43]	20	0.88–0.95	Reported
	AFSS	[22,26,27]	14	0.94	Reported
	AFQLQ	[41]	26	0.78–0.89	Reported
Cardiac-specific	NHP	[32]	45	0.72	Reported
	IIRS	[22]	13	0.88	Reported
	QLI-CV	[39]	35	0.94–0.95	Reported
	SCL	[22,27]	16	0.84–0.91	Reported
	SDQL	[23]	8	Reported	Reported
Generic	SF-12	[36]	12	0.89 (PCS) and 0.86 (MCS)	Reported
	SF-36	[22,24,25,26,27,28,29,30,31,33,37,38,39,40,44]	36	0.89–0.93 (PCS) and 0.84–0.88 (MCS)	Reported
	SAS	[22,27]	20	0.62 **	Reported
	EQ-5D	[40]	5	0.70	Reported
	EQ-VAS	[40]	1	NA	Reported

* Cronbach alpha. ** Weighted kappa statistic for reproducibility. AFEQT: Atrial fibrillation effect on quality of life; AFSC: Atrial fibrillation symptom checklist; AFQLQ: Atrial fibrillation quality of life questionnaire; EQ-5D: Euroqol-5d; EQ-VAS: Euroqol-visual analog scale; IIRS: Illness intrusiveness rating scale; NA: Not applicable; NHP: Nottingham health profile; QLI-CV: Quality of life index-cardiac version; SAS: Specific activity scale; SCL: Symptom checklist; SDQL: Scale of disease and quality of life; SF-12: 12-item short form survey, SF-36: Short form 36 health survey.

**Table 3:** Factors affecting HRQoL in patients with AF according to Ferrans and colleagues’ conceptual model.

Domain (No. of Predictors/Articles)	Factors (Frequency)	Article Number(s)	Significance
Biological function	Duration of illness (2)	[22,38]	–/+++
(9/7)	Left ventricle ejection fraction (LVEF) (1)	[22]	–
	Left atrial dimension (1)	[22]	–
	Stroke of comorbidity (1)	[29]	++
	Type of AF (1)	[32]	+
	Heart rate (1)	[33]	+
	CHADS2 score (1)	[36]	+++
	Brain natriuretic peptide (BNP) level (1)	[40]	++
	LV systolic and diastolic function (1)	[40]	+++
Symptoms	Anxiety (4)	[23,26,31,38]	++/++/++/+++
(5/9)	Symptom frequency and severity (4)	[22,29,35,43]	+/+++/NA/+++
	Depression (2)	[32,40]	++/+++
	Perceived stress (1)	[31]	+++
	Uncertainty (1)	[38]	+++
Functional status	NYHA class (3)	[22,29,38]	+/+++/+++
(2/4)	Exercise performance (1)	[27]	++
General health	Fear of AF attack (1)	[23]	++
perceptions	Illness perception/identity (1)	[31]	+
(9/4)	Emotional distress including anxiety and fear of stroke (2)	[34,42]	Qualitative studies
	Feeling uninformed and unsupported (2)	[34,42]	Qualitative studies
	Acceptance of the disease (2)	[34,42]	Qualitative studies
	Positive coping with living with AF (2)	[34,42]	Qualitative studies
	Adverse effect of social life (1)	[42]	Qualitative studies
	Inability to carry out daily living activities (1)	[42]	Qualitative studies
	Sexual problem (1)	[42]	Qualitative studies
Characteristics of	Exercise intervention (2)	[28,37]	++/+++
the individual	Gender (3)	[25,30,32]	+/+/+
(8/9)	Age (2)	[29,38]	++/+++
	Alcohol use (1)	[29]	+++
	Sleep (1)	[25]	++
	Employment (1)	[29]	+
	Optimism (1)	[40]	++
	Neuroticism (1)	[24]	++
Characteristics of	Significant others (1)	[38]	+++
the environment (2/1)	Financial burden (1)	[38]	+++

+: *p* < 0.05; ++: *p* < 0.01; +++: *p* < 0.001; NA: Not applicable to study; AF: Atrial fibrillation; CHADS_2_: Congestive heart failure history, hypertension history, age ≥75 years, diabetes mellitus history, stroke or transient ischemic attack symptoms previously; NYHA class: New York Heart Association functional class.
